# Retinoic acid mitigates the NSC319726-induced spermatogenesis dysfunction through cuproptosis-independent mechanisms

**DOI:** 10.1007/s10565-024-09857-6

**Published:** 2024-05-01

**Authors:** Haisheng Yi, Tong Chen, Guitian He, Lingyun Liu, Jiantao Zhao, Kaimin Guo, Yin Cao, Penghao Sun, Xu Zhou, Boqi Zhang, Chunjin Li, Hongliang Wang

**Affiliations:** 1 Department of Andrology, The First Hospital of Jilin University, Jilin University, Changchun, 130012 China; 2 College of Animal Sciences, Jilin University, Changchun, 130062 China

**Keywords:** Copper ionophore, NSC319726, Spermatogenesis dysfunction, Cuproptosis, Retinoic acid, Adjuvant medication

## Abstract

Copper ionophore NSC319726 has attracted researchers' attention in treating diseases, particularly cancers. However, its potential effects on male reproduction during medication are unclear. This study aimed to determine whether NSC319726 exposure affected the male reproductive system. The reproductive toxicity of NSC319726 was evaluated in male mice following a continuous exposure period of 5 weeks. The result showed that NSC319726 exposure caused testis index reduction, spermatogenesis dysfunction, and architectural damage in the testis and epididymis. The exposure interfered with spermatogonia proliferation, meiosis initiation, sperm count, and sperm morphology. The exposure also disturbed androgen synthesis and blood testis barrier integrity. NSC319726 treatment could elevate the copper ions in the testis to induce cuproptosis in the testis. Copper chelator rescued the elevated copper ions in the testis and partly restored the spermatogenesis dysfunction caused by NSC319726. NSC319726 treatment also decreased the level of retinol dehydrogenase 10 (RDH10), thereby inhibiting the conversion of retinol to retinoic acid, causing the inability to initiate meiosis. Retinoic acid treatment could rescue the meiotic initiation and spermatogenesis while not affecting the intracellular copper ion levels. The study provided an insight into the bio-safety of NSC319726. Retinoic acid could be a potential therapy for spermatogenesis impairment in patients undergoing treatment with NSC319726.

**Supplementary Information:**

The online version contains supplementary material available at 10.1007/s10565-024-09857-6.

## Introduction

Copper ionophores have garnered significant interest in materials research, environmental science, chemistry, and biomedical research (Steinbrueck et al. 2020; Vareda et al. 2021). In biomedical research, they are used for delivering metal ions in a tissue-specific manner as liver-targeted copper supplementations (Su et al. 2018), novel antimicrobial in drug-resistant pathogenic bacteria (O’Brien et al. 2023), positron emission tomography imaging agents (Petersen et al. 2011), uveal melanoma mutant specific inhibitors (Li et al. 2022), and promising antineoplastic drugs (Jiang et al. 2022; Shanbhag et al. 2021; Yang et al. 2023). Given their significant in vivo application potential, the toxicology of copper ionophores should be evaluated before any clinical use.

More recently, scientists discovered that copper ionophores—such as elsclomol, disulfiram, and NSC319726—could cause iron-sulfur cluster protein loss and mitochondrial lipoylated protein aggregation. These findings were characterized as a novel cuproptosis mechanism that differed from the other forms of cell death that are currently understood (Tsvetkov et al. 2022). The discovery of cuproptosis again attracted considerable interest in the potential applications of copper ionophores. Therefore, evaluating their toxicity requires further extensive investigation, and assessing their reproductive toxicity is also essential during the risk assessment of chemicals. Progress in utilizing ionophores in therapeutic settings is hindered until their biosafety concerns are adequately resolved. Preclinical findings from a phase II clinical trial indicated that elesclomol was well-tolerated in patients with ovarian, fallopian tube, or primary peritoneal cancer and metastatic melanoma. However, the potential reproductive toxicity of the drug has not been extensively studied (O’Day et al. 2009). Studies showed that disulfiram slightly impacted female reproduction (Helmbrecht and Hoskins 1993; Kelty et al. 2021; Nora et al. 1977; Teng et al. 2023; Thompson and Folb 1985), while disulfiram affected male reproduction by inhibiting retinaldehyde dehydrogenase activity and spermatogenesis (Cho et al. 2021; Hoover et al. 1991). However, the reproductive toxicity of NSC319726 has not been reported yet.

NSC319726 holds excellent potential for clinical applications. It is a member of the thiosemicarbazone group of metal ion chelators, which bind to zinc, copper, iron, and magnesium, among other divalent metals. NSC319726 (ZMC1) is a zinc metallochaperone that, depending on its redox potential and zinc ion chelating properties, can restore the wild-type structure and function of the p53R^175^ mutant in order to inhibit tumor growth. However, the p53^R172H/R172H^ mice were vulnerable to death due to the toxicity of NSC319726 (Yu et al. 2017; Yu et al. 2012). NSC319726 can inhibit ribosomal biogenesis and show anti-fungal and antibacterial activities against resistant strains of *Escherichia coli* (Li et al. 2021; Lin et al. 2015; Sadaka et al. 2018; Sun et al. 2017). NSC319726 has excellent potential for the treatment of glioblastoma, ovarian cancer, and colorectal cancer (Berkel and Cacan 2020; Hashemi et al. 2022; Shimada et al. 2018; Xue et al. 2015). The biosafety of NSC319726 should be evaluated, particularly regarding reproductive toxicity, due to its numerous potential clinical uses in the biomedical area.

NSC319726 predominantly exerted biological activity by transporting divalent metal ions, mainly copper, inside the cells. Copper can severely impair spermatogenesis by directly altering oxidative stress state and autophagy levels in the testis (Guo et al. 2021; Guo et al. 2022), suggesting the ability of copper ionophore to damage spermatogenesis. The current study aimed to investigate whether NSC319726 could affect mice spermatogenesis. It is necessary to comprehensively evaluate the reproductive toxicology of copper ionophore NSC319726 before its clinical use.

## Experimental methods

### Materials and reagents

NSC319726 (PubChem CID: 5,351,307) was purchased from MedChemExpress (Cat. HY-18634). Ammonium tetrathiomolybdate (TTM) was purchased from Sigma-Aldrich (Cat. 323,446). Retinoic acid was obtained from Meilunbio (Cat. 302–79-4).

The study utilized the following antibodies: rabbit anti-DDX4 (Bioword, BS72725, IF:1:200, WB:1:1000), mouse anti-SYCP3 (Abcam, ab97672, IF:1:200, WB:1:1000), rabbit anti-CYP17A1 (Bioword, MB10800, WB:1:1000), rabbit anti-ZO-1 (Bioword, BS71522, WB:1:1000), rabbit anti-Vinculin (Bioword, BS62273, WB:1:1000), rabbit anti-β-Catenin (Abclonal, A11932, WB:1:1000), mouse anti-GAPDH (Bioword, MB001, WB:1:1000), rabbit anti-DLAT (Proteintech,13,426–1-AP, 1:2000), rabbit anti-LIAS (Proteintech, 11,577–1-AP, 1:1000), rabbit anti-FDX1 (Bioword, BS71332, WB:1:1000), rabbit anti-STRA8 (OmnimAb, OM292470, IF:1:200, WB:1:1000), and rabbit anti-RDH10 (Affinity Biosciences, DF12105, IF:1:200, WB:1:1000).

### Ethics statement

The rodents were allowed to adjust to the laboratory settings for one week at a temperature of 22 ± 2℃ and humidity of 55 ± 5%, with water and food freely available. All animal-related research has been authorized by the Experimental Animal Ethics Committee of Jilin University, China, with the approval number SY202312009.

### Animals and treatments

Male ICR mice that were eight weeks old were acquired from Jilin University's Experimental Animal Center (Jilin University, Changchun, China). All the mice were weighed and randomized into subgroups by generating a random number. The groups consisted of a control group (n = 8 mice were injected intraperitoneally with solvent daily) and an NSC319726 group (n = 8 mice were injected intraperitoneally with 1 mg/kg of NSC319726 daily for five weeks).

To treat testis damage induced by NSC319726, the mice were co-treated with copper chelator tetrathiomolybdate (TTM) and retinoic acid (RA). The healthy male ICR mice (8-week-old) were arbitrarily separated into four groups (n = 8). The control group mice were injected with the control solvent intraperitoneally daily for 10 weeks. The NSC group mice were intraperitoneally injected with 1 mg/kg NSC319726 daily for 10 weeks. For 10 weeks, the mice in the TTM/RA group received intraperitoneal injections of 1 mg/kg NSC319726 daily. In the final five weeks, the mice were administered intraperitoneal injections of either 5 mg/kg RA or 0.03 mg/mL of TTM in their drinking water. Samples were collected from all the mice for the follow-up analysis after euthanasia.

### Sample collection and processing

Following tribromoethanol anesthesia, all of the mice were euthanized in accordance with the approved methodology of Jilin University's Experimental Animal Ethics Committee in order to collect samples. Cardiac blood sampling were performed after anesthesia. The mice were weighed weekly, and their testes were collected and weighed after euthanasia. The testis index was calculated as weight in grams per gram of body weight. The epididymis was suspended in PBS buffer under 37℃, and the sperm counts, and malformation degree were measured as described previously by Seed J et al. (1996). A part of the samples was prefixed in a 4% paraformaldehyde solution and used for histological/fluorescence analysis, while the remaining part was processed and used for molecular analysis.

### Fertility

After the 5-week treatment, male and ten-week-old female mice (at a ratio of 1:3) in each group were mated for 2 weeks. The vaginal plugs were examined daily for the purpose of identifying timed pregnancies. The number of pregnant mice and suckling mice was recorded for each group, and the pregnancy rate was calculated.

### Histopathological section and immunofluorescence analysis

The tissues of the testes and epididymis were immersed in paraffin, segregated, fixed in 4% paraformaldehyde, and stained with hematoxylin and eosin (HE). The tissue slices were first exposed to primary antibodies for immunofluorescence at 4°C for a whole night. Subsequently, the tissue sections were treated for 1 h at room temperature with secondary antibodies labeled with FITC. The slices were mounted in DAPI-containing media and examined under a fluorescence microscope. Images were quantified and analyzed using Image J.

### Biochemical assays

The harvested testes were weighed and homogenized with PBS. After letting the whole blood samples sit at 37 °C for 30 min, they were centrifuged at 3000 rpm for 15 min to collect serum. Testosterone levels were quantified using an ELISA kit (Elabscience, E-OSEL-M0003, China) following the manufacturer's guidelines. The BCA kit assessed the total amount of protein in samples to quantify and normalize test findings. The retinol and retinoic acid levels were determined using their respective commercially available ELISA kits following the manufacturer's protocol (Cusabio, CSB-E07891m, Wuhan) (Sakashita et al. 2022).

#### *Examining the integrity of the blood-testis barrier *in vivo

Using biotin, the blood–testis barrier (BTB) integrity assay was conducted as previously reported (Huang et al. 2021; Jia et al. 2017). After the 5-week treatment, 50 μL EZ-Link Sulfo-NHS-LC-Biotin (Thermo Fisher Scientific, Cat. 21,338) was injected into the testis interstitium of mice. The testes were extracted after 40 min in preparation for cryosection at -20 °C. After blocking the 10 µm sections of testis tissue with 5% bovine serum albumin, they were incubated for 2 h with Alexa Fluor 568-conjugated streptavidin (AAT Bioquest, Cat. 16,960). After mounting with a DAPI medium, the tissue sections were observed under a fluorescence microscope. Blood–testis barrier integrity was analyzed quantified by the mean fluorescence intensity in seminiferous tubule.

### Copper ion level detection

Using a Copper Colorimetric Assay Kit (E-BC-K300-M/E-BC-K775-M, Elabscience) and the manufacturer's guidelines, the testes and cell copper ion levels were determined (Xie et al. 2022). After measuring the absorbance at 580 nm, the standard curve was used to determine the copper levels.

### Transcriptomic analysis

Thermo Fisher, CA, USA's TRIzol reagent (15,596,018) was used to extract total RNA, and Dynabeads Oligo (dT) was used to purify the mRNAs (5 µg) from the extract. Subsequently, SuperScriptTM II Reverse Transcriptase (Invitrogen, cat. 1,896,649, USA) was used to split the mRNA into brief segments for reverse transcription into cDNA. Finally, the Illumina NovaseqTM 6000 platform (LC-Bio Technology CO., Ltd., Hangzhou, China) was utilized to conduct the 2 × 150 bp paired-end sequencing (PE150) in accordance with the guidelines provided by the manufacturer. The R programs DESeq2 and edgeR were used for the DEG analysis comparing the two groups. Differentially expressed genes (DEGs) were defined as those having an absolute fold change of ≥ 2 and a P-value of < 0.05. The Kyoto Encyclopedia of Genes and Genomes (KEGG) pathways and Gene Ontology (GO) functions were examined for enrichment.

#### Metabolomic analysis

The metabolites were identified by profiling the extraction supernatants using liquid chromatography-mass spectrometry (LC–MS) after they were extracted with 80% methanol buffer. A Q-Exactive high-resolution tandem mass spectrometer (Thermo Scientific), which functioned in both positive and negative ion modes, was used to identify the metabolites that eluted from the column. XCMS software was used to process the MS data. The mass-to-charge ratio (m/z) and specific retention time (RT) of each metabolite were used to identify it. The KEGG database was utilized to annotate the metabolites through a comparison between the precise molecular mass data (m/z) of the samples and the information contained in the database. The student t-tests were applied to identify variations in metabolite levels between the two groups. Multiple tests were accounted for in the P-value using the Benjamini–Hochberg false discovery rate (FDR) method.

#### Real-time quantitative PCR

Total RNA from testicles was extracted using TRIzol reagent (Invitrogen) according to the manufacturer's guidelines. Using an All-in-One 5X RT MasterMix Kit (Aibimeng Biotechnology Co., Ltd, China), 1 μg of total RNA was converted into complementary DNA (cDNA). Gene expression was detected with SYBR Green and evaluated quantitatively using the 2^−ΔΔCT^ technique. PPIA was utilized as an internal reference (Gong et al. 2014). All experiments were replicated three times independently.

The following primer sequences were used:

Mus-CYP17A1;

F: CATCTCATTACACCCACACCC,

R: CACATCAAAGTCAAACCTCTGC.

Mus-ZO-1;

F: GGGGCCTACACTGATCAAGA,

R: TGGAGATGAGGCTTCTGCTT

Mus- Vinculin;

F: TGGTCTAGCAAGGGCAATGA

R: CTCGTCACCTCATCAGAGGC

Mus-β-catenin;

F: CAGATCCCATCCACGCAGTT

R: ATTGCACGTGTGGCAAGTTC.

### Western blotting

Using RIPA lysis buffer combined with proteinase inhibitor and phosphatase inhibitor from Solarbio Life Sciences, the protein was extracted from testicular tissue. Protein samples were separated on a 10% SDS-PAGE gel, and the amount of protein was quantified using Beyotime Biotechnology's BCA Protein Assay Kit. After the proteins on the gel were separated, they were moved to a PVDF membrane, blocked for an hour at room temperature using 5% milk in TBST, and then incubated with primary antibodies for an additional night at 4 °C. After that, the membrane was treated for 1 h at room temperature with the secondary antibody, goat anti-mouse/rabbit-IgG (Bioworld, BS12478/BS13278, 1:5000 dilution), coupled with horseradish peroxidase (HRP). Shanghai Tanon Technology's Tanon-5200 fully automated digital gel imaging analysis equipment was used to detect the protein bands by enhanced chemiluminescence (ECL). Subsequently, the bands were examined using ImageJ software.

### Cell culture and cell viability assay

We obtained the cell lines utilized in this investigation from the laboratory cell bank. The media used to cultivate TOV112D cells was M199:MCDB105 (1:1) supplemented with 15% FBS. HCT116 cells were cultured in 10% FBS-containing McCoy's 5A medium. A 1 M concentration of NSC319726 was applied to the cells. The viability of the cells was assessed utilizing the Cell Counting Kit-8 (CCK-8; Dojindo). The cells were dispersed into 96-well plates, and retinoic acid, or NSC319726, was applied to each well. The cell viability was determined following the manufacturer's guidelines by calculating absorbance at 450 nm.

### Statistical analyses

Every experiment was conducted at least three times. The two-tailed Student's t-test was utilized to assess the statistical differences between the two groups. On the other hand, for multigroup comparisons, one-way analysis of variance (ANOVA) with a post-test was utilized to investigate differences between more than two groups (such as Tukey or Duncan tests).

The *P*-values < 0.05, < 0.01, < 0.001 indicated statistical significance, while ns indicated no significance.

## Results

### NSC319726 impaired male reproductive system

**Fig. 1:**
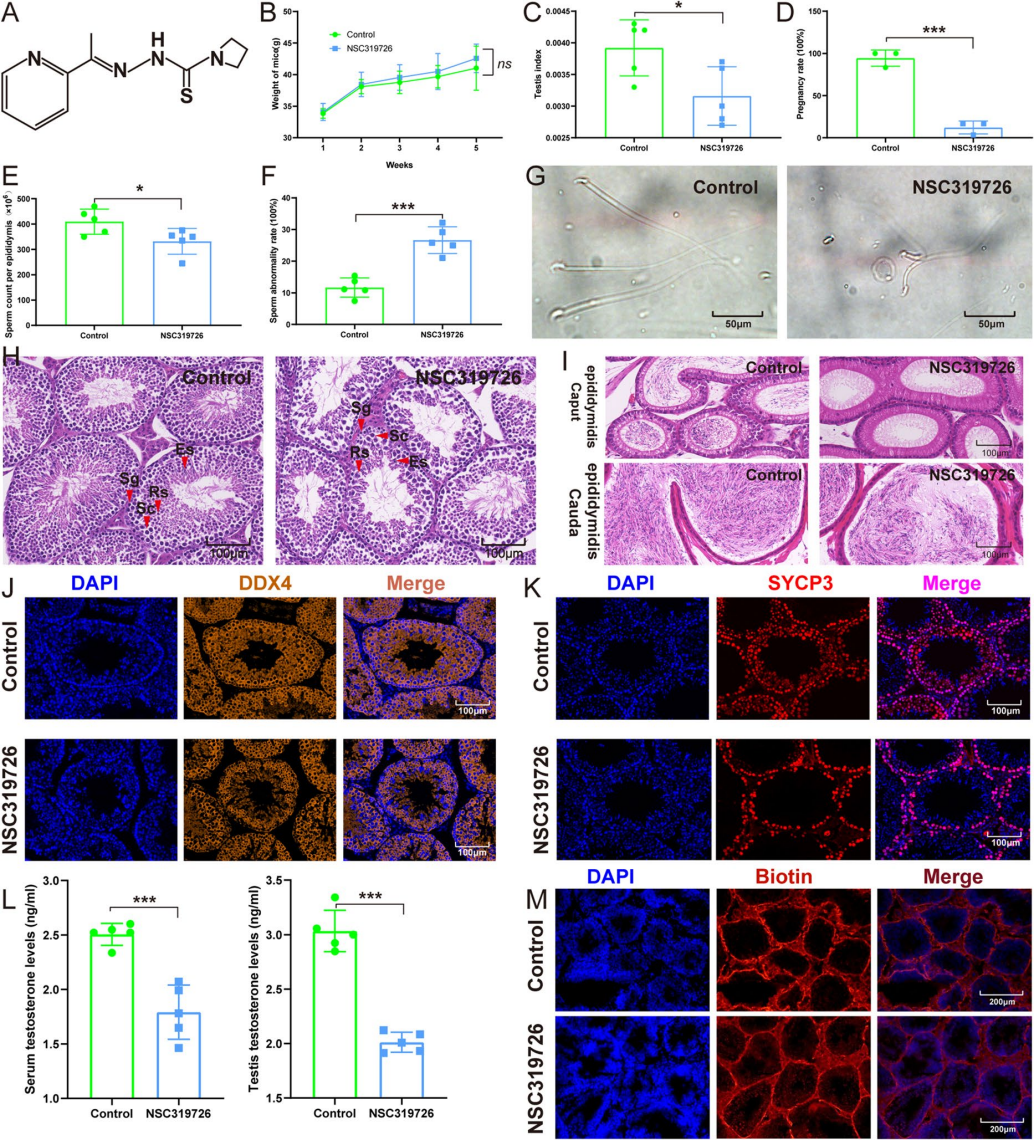
NSC319726 impaired male reproductive system. (**A**) The structural formula of NSC319726. (**B**) Mice body weights (n = 5). (**C**) Testis index (n = 5). (**D**) Pregnancy rate (n = 3). (**E**) Sperm count per epididymis (n = 5). (**F**) Sperm malformation rate (n = 5). (**G**) Typical morphology of sperm in both groups. (**H**) Hematoxylin–eosin staining of histopathological changes in the testis. (Es, elongated spermatid; Rs, round spermatid; Sc, spermatocyte; red arrow: Sg, spermatogonia). (**I**) Hematoxylin–eosin staining of histopathological changes in the epididymis. (**J**) Immunofluorescence staining of DDX4 in testis. (**K**) Immunofluorescence staining of SYCP3 in testis. (**L**) Testosterone levels in serum and testis tissue. (**M**) Biotin tracer assay. *P < 0.05, **P < 0.01, and ***P < 0.001. ns, not significant

In comparison to the control group, there was no significant variation in the body weight of mice following treatment with NSC319726 (Fig. 1A and Fig. 1B). At the same time, the testis index and pregnancy rate decreased significantly (Fig. 1C-1D). Moreover, there was a decrease in sperm counts with an increased sperm abnormality rate after the NSC319726 treatment (Fig. 1E-1G). Hematoxylin–eosin (HE)-stained testis slices were subjected to a histological analysis, which demonstrated that the NSC319726 group showed constriction of the tubule and lumen of the tubule and a decrease in the thickness of the seminiferous epithelium in compared with the control group (Fig. 1H, Fig. S1A-C). HE staining demonstrated a reduction in the quantity of sperm in the caput and cauda epididymis of the NSC319726 group compared to the control group (Fig. 1I).

To ascertain germ cell injury, immunofluorescence analysis was conducted utilizing antibodies targeting spermatocyte-specific marker SYCP3 (Nagamori et al. 2011) and pan-germ cell marker DDX4 (Miao et al. 2021). The immunofluorescence analysis showed a decrease in the total germ cells and spermatocytes after the NSC319726 treatment ((Fig. 1J-K and S1D-E).

Apart from directly impairing spermatogenesis, NSC319726 also affected the androgen levels and the integrity of the blood testis barrier. The NSC319726 group exhibited significantly lower testosterone levels in serum and testis tissues than in the control group (Fig. 1L). Through the biotin tracer experiment, it was found that NSC319726 weakened the blood testis barrier (Fig. 1M and S1F).

### The characterization of testis metabolome after the NSC319726 treatment

**Fig. 2:**
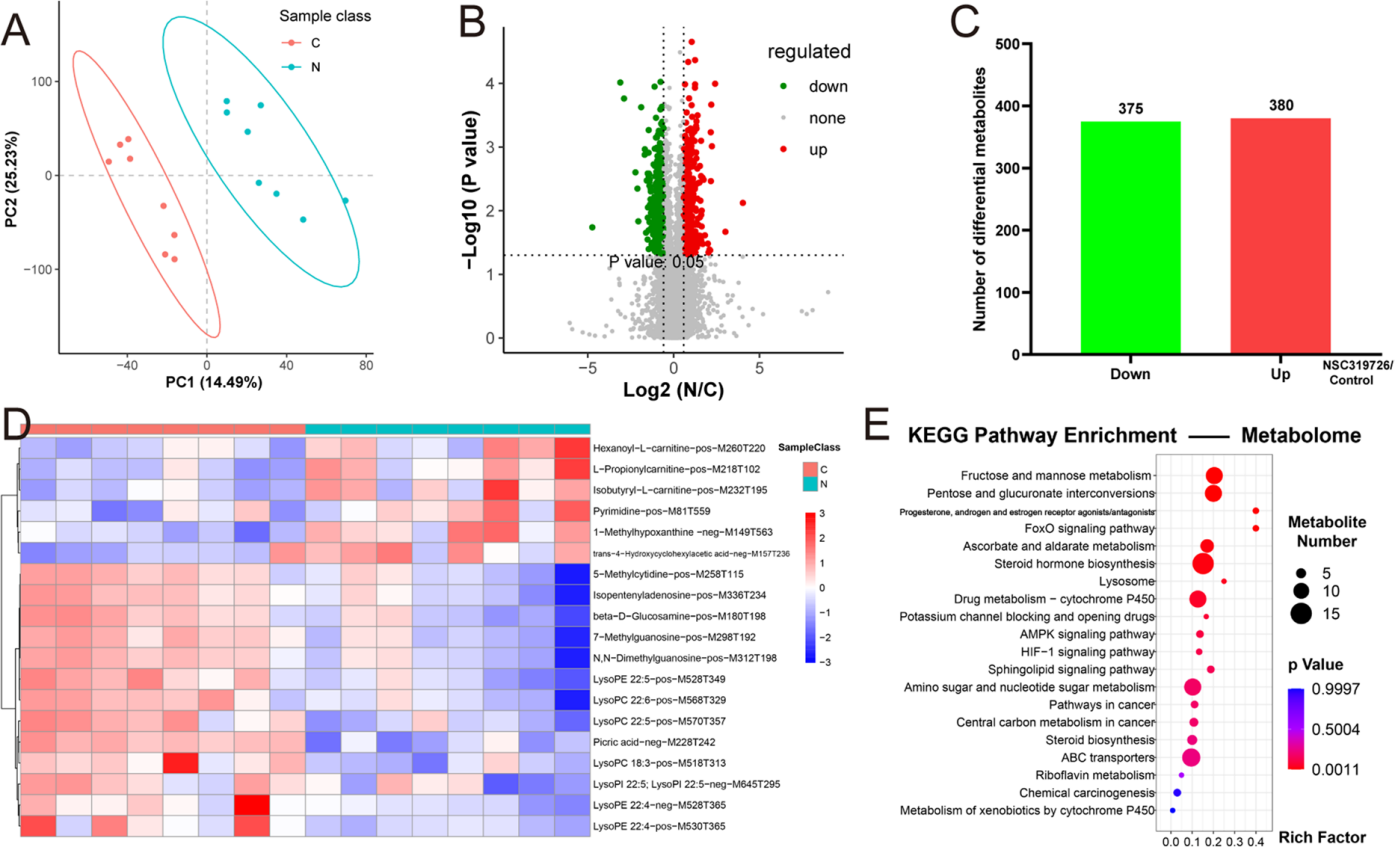
Characterization of testis metabolome after NSC319726 treatment. (**A**) The PCA plot of the metabolome in testis (n = 8). (**B**) Volcano plot of the differential metabolite ions. (**C**) The number of differential metabolites. (**D**) Heatmaps of secondary metabolites in different groups. (**E**) KEGG pathway enrichment analyses of the differential metabolites

Non-targeted metabolomic was used to determine the impact of NSC319726 on the testis function (n = 8). The treatment of mice with NSC319726 drastically changed their metabolic profile, as shown in Fig. 2A. A comprehensive set of 17,131 differential metabolites was detected in the NSC319726 group compared to the control group. Among these, 375 were downregulated, and 380 were upregulated (P < 0.05; log2-fold ratio > 2 or < 0.5; Fig. 2B and 2C). Further analysis showed that among these differential metabolites, six metabolites (Hexanoyl − L − carnitine, L − Propionylcarnitine, Isobutyryl − L − carnitine, Pyrimidine, 1-methyl hypoxanthine, and trans − 4 − Hydroxycyclohexylacetic acid) increased. In comparison, thirteen metabolites (5-methylcytidine, Isopentenyladenosine, beta-d-glucosamine, 7 − Methylguanosine, N, N − Dimethylguanosine, LysoPC 18:3, Picric acid, LysoPC 22:5, LysoPC 22:6, LysoPE 22:5, LysoPI 22:5; LysoPI 22:5, LysoPE 22:4, LysoPE 22:4) decreased in the NSC319726 group (Fig. 2D). The steroid hormone biosynthesis and FoxO signaling pathway exhibited a notable enrichment of distinct metabolites between the two groups (Fig. 2E).

The FoxO signaling pathway is essential for maintaining and initiating spermatogenesis in rodent spermatogonial stem cells. Enriching metabolites in the FoxO signaling pathway indicated that NSC319726 might disturb the maintenance of spermatogonial stem cells and initiation of spermatogenesis.

### The characterization of testis transcriptome after the NSC319726 treatment

**Fig. 3:**
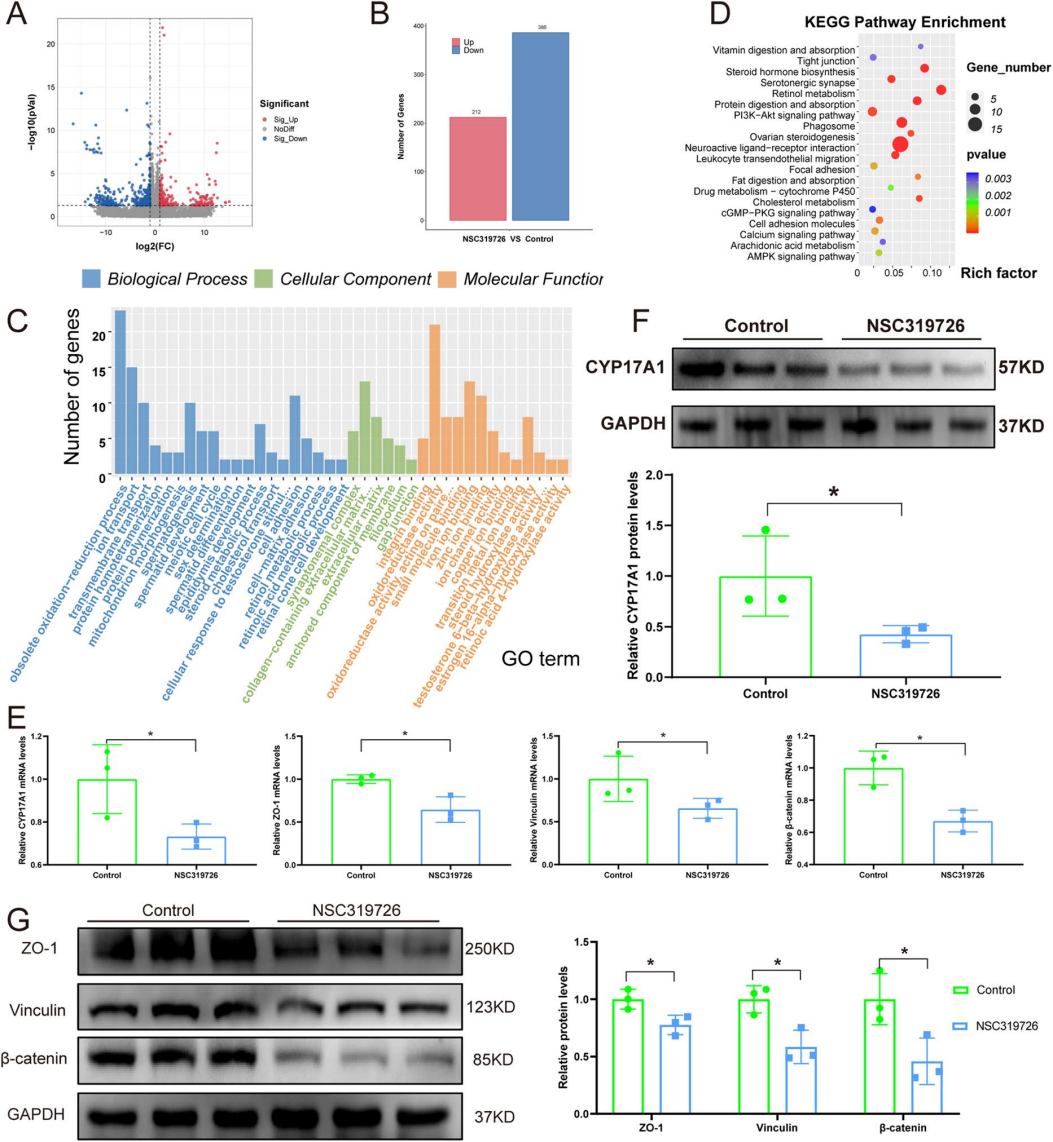
Characterization of testis transcriptome after the NSC319726 treatment. (**A**) The testis transcriptome's genes with differential expression (DEGs) are plotted in a volcano. (**B**) Number of DEGs. (**C**) GO enhancement analyses of the DEGs. (**D**) KEGG enrichment analyses of the DEGs. (**E**) mRNA expression levels of CYP17A1, ZO-1, Vinculin, and β-catenin. (**F**) Western blot of CYP17A1 (n = 3). (**G**) Western blot of ZO-1, Vinculin, and β-catenin (n = 3). *P < 0.05

To explore the possible mechanisms of damage to the testis caused by NSC319726, RNA sequencing analysis of testis tissues from the control and NSC319726 group (n = 3 per group) was performed. In comparison to the control group, the NSC319726 group exhibited 212 upregulated genes and 386 downregulated genes with statistical significance (P < 0.05, log2-fold change > 2 or < 0.5) (Fig. 3A-3B). GO and KEGG enrichment analysis of the differential genes indicated that the DEGs were enriched in transition metal ion binding and transportation, spermatid development, steroid synthesis, epididymal development, tight junctions, and retinoic acid metabolic (Fig. 3C-3D).

Employing qPCR, the mRNA expression levels of key genes involved in androgen production and the tight junction of the blood-testis barrier were confirmed. The outcomes demonstrated that following NSC319726 administration, the mRNA expression levels of CYP17A1, ZO-1, Vinculin, and β-catenin were significantly downregulated (Fig. 3E). The protein expression of CYP17A1, ZO-1, Vinculin, and β-catenin also decreased, indicating the impairment of androgen biosynthesis and integrity of blood-testis barrier in testes after the NSC319726 treatment (Fig. 3F-3G).

### Cuproptosis inhibition partly reversed NSC319726-induced spermatogenesis dysfunction

**Fig. 4:**
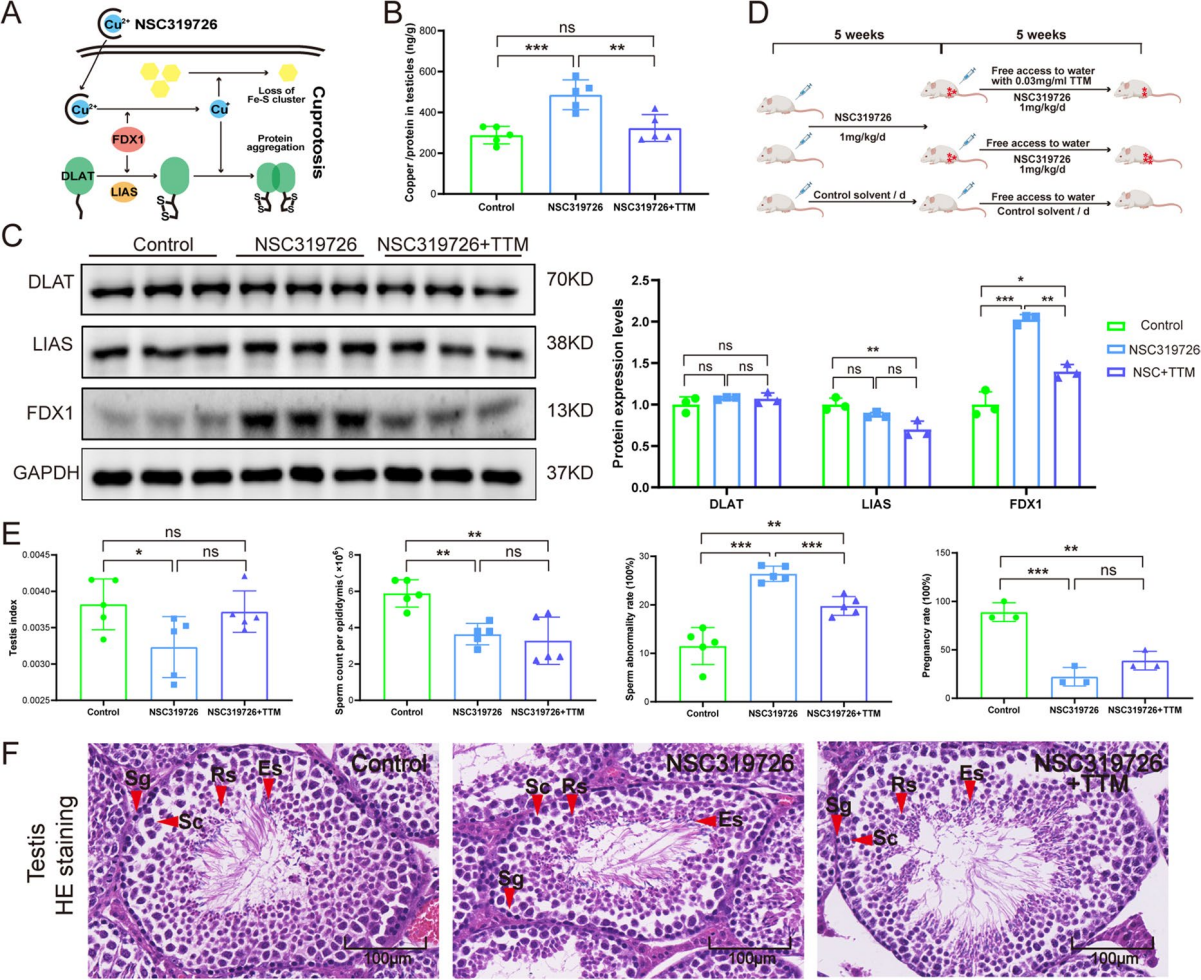
Inhibition of cuproptosis partly reversed the spermatogenesis dysfunction caused by NSC319726. (**A**) Schematic representation of cuproptosis. (**B**) Copper levels in testes (n = 5). (**C**) Western blot of DLAT, LIAS, and FDX1 in cuproptosis (n = 3). (**D**) Flowchart of medication administration. (**E**) Testis index, sperm count per epididymis, sperm malformation, and pregnancy rate. (**F**) HE staining of testis tissues. (red arrow: Es, elongated spermatid; Rs, round spermatid; Sc, spermatocyte; Sg, spermatogonia)

Studies have indicated that NSC319726 can transport copper ions into cells and trigger cuproptosis (Fig. 4A). Copper ion levels were measured to investigate the possibility of cuproptosis in the testis. According to the data, the testicular concentration of copper ions and the expression levels of FDX1, a crucial protein involved in cuproptosis, were significantly increased following NSC319726 therapy. This indicated cuproptosis occurred in the testis after NSC319726 treatment (Fig. 4B and 4C). To further explore whether cuproptosis promoted the spermatogenesis dysfunction after NSC319726 treatment, the mice were treated with copper chelators TTM after NSC319726 treatment (Fig. 4D). Results showed that TTM rescued the elevated copper levels and expression of FDX1 in testis (Fig. 4B and 4C). TTM also partly recovered the decreased testis index, pregnancy rate, and sperm count as well as the elevated sperm malformation rate caused by NSC319726 (Fig. 4E). HE-stained testis tissue sections showed that TTM partly repaired the reduced germs and total germ cells in seminiferous tubule caused by NSC319726 (Fig. 4F). The TTM treatment restored the increased protein levels of FDX1 in cuproptosis, as determined by Western blot analysis (Fig. 4C). The findings of this study suggest that cuproptosis contributed to the impairment of spermatogenesis induced by NSC319726.

### Retinoic acid can rescue the spermatogenesis dysfunction caused by NSC319726

**Fig. 5:**
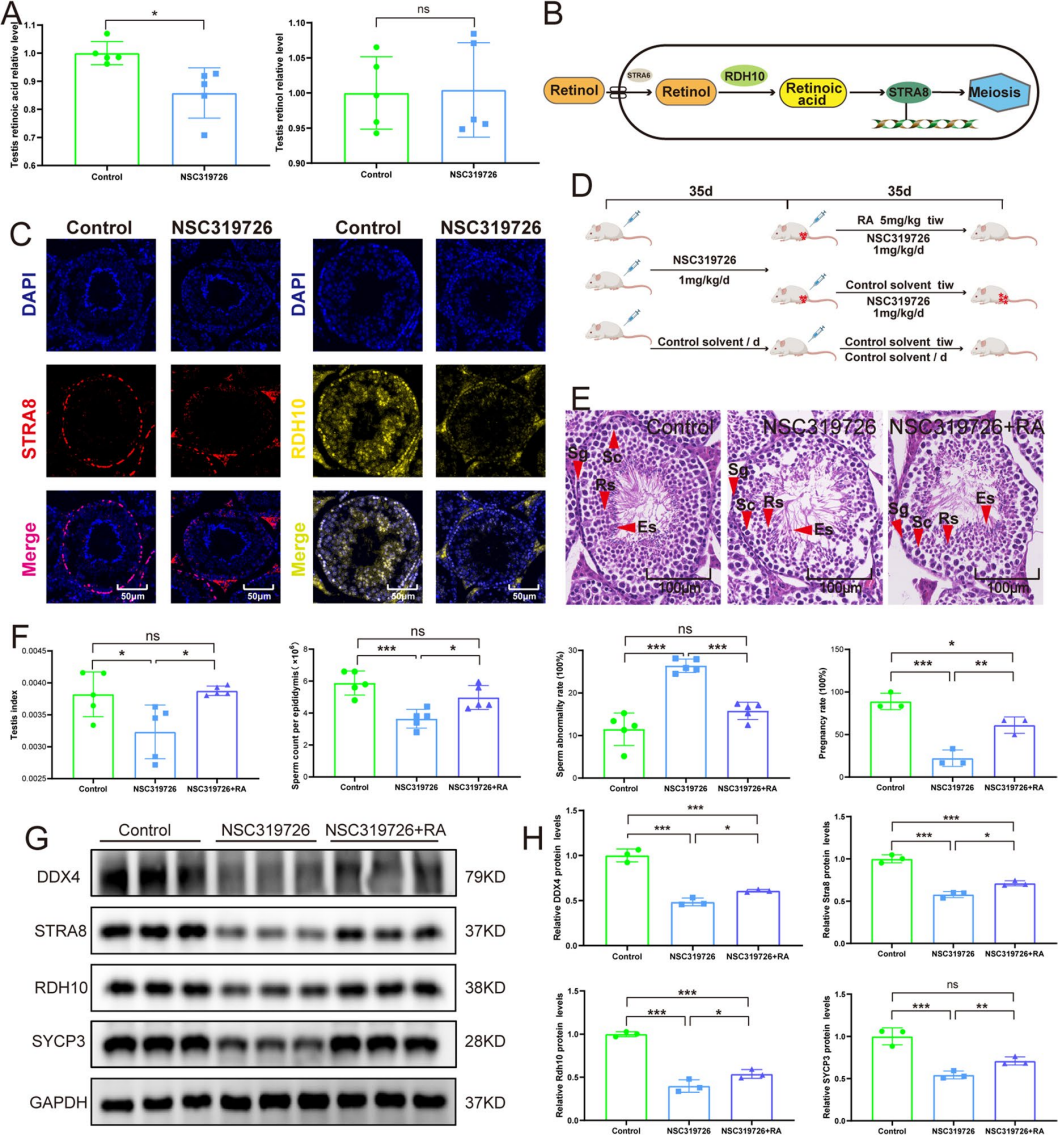
Retinoic acid could rescue the spermatogenesis dysfunction caused by NSC319726. (**A**) Retinol and retinoic acid levels in testis (n = 5). (**B**) Schematic of retinoic acid initiating meiosis. (**C**) Immunofluorescence staining of STRA8 and RDH10 in testis. (**D**) Flowchart of medication administration. (**E**) HE staining of testis tissue sections. (red arrow: Es, elongated spermatids; Sg, spermatogonia; Rs, round spermatid; Sc, spermatocyte). (**F**) Testis index, sperm count per epididymis, sperm malformation, and pregnancy rate. (**G**) Western blot of DDX4, STRA8, RDH10, and SYCP3 in spermatogenesis (n = 3). (**H**) Quantification of Western blot gray-scale

Immunofluorescence analysis showed decreased spermatocyte and total germ cells after the NSC319726 treatment (Fig. 1J and 1K). The enrichment of the FoxO signaling pathway in the metabolome suggested that NSC319726 inhibited both the initiation and maintenance of spermatogonial stem cells (Fig. 2E). The enrichment analyses of KEGG pathways and GO terms indicated that retinoic acid metabolism was crucial following NSC319726 treatment (Fig. 5A). Retinoic acid is vital in kickstarting spermatogenesis, creating the blood-testis barrier, and guiding spermatogonial differentiation (Schleif et al. 2022). The disorder of retinoic acid metabolism might be a pivotal contributor to the spermatogenesis dysfunction caused by NSC319726.

To further confirm whether NSC319726 was relevant to retinol metabolism in spermatogenesis, the contents of retinol and retinoic acid in the testis were measured. After NSC319726 treatment, the testis retinoic acid level showed a significant decrease. In contrast, the testis retinol level exhibited no apparent changes (Fig. 5A). Under physiological conditions, retinol is metabolized into its active form, retinoic acid, by retinol dehydrogenase 10 (RDH10) in the testis. Retinoic acid can trigger meiotic entry by promoting the expression of the *STRA8* gene (stimulated by retinoic acid 8, Fig. 5B) (Griswold 2016). Immunofluorescence analysis revealed a notable reduction in the STRA8 and RDH10 levels after treatment with NSC319726 (Fig. 5C and S1G). Testis retinol level showed no apparent changes after NSC319726 treatment, which indicated NSC319726 did not affect the transportation of retinol into the cell. It was speculated that NSC319726 influenced the intracellular conversion of retinol to retinoic acid.

To investigate whether retinoic acid could rescue the spermatogenesis dysfunction, the mice were treated with retinoic acid after NSC319726 treatment (Fig. 5D). The HE-stained testis tissue sections showed that retinoic acid could rescue the reduction in the total germ cells and spermatocyte caused by NSC319726 (Fig. 5E). Results showed that retinoic acid significantly restored the decrease in testis index, pregnancy rate, and sperm count as well as the elevated sperm malformation rate caused by NSC319726 (Fig. 5F). Western blot analysis indicated that retinoic acid also restored the decreased protein levels of STRA8, RDH10, DDX4, and SYCP3 after NSC319726 treatment (Fig. 5G and 5H). Moreover, the increased protein expression level of STRA8 indicated the rescue of meiotic entry, and the increased protein expression levels of DDX4 and SYCP3 showed the rescue of total germ cells and spermatocytes. Therefore, it was speculated that NSC319726 led to spermatogenesis dysfunction by inhibiting the expression of RDH10 and inhibiting the meiotic entry. Retinoic acid showed better treatment effects than TTM, suggesting its predominant role in the spermatogenesis dysfunction caused by NSC319726.

### Retinoic acid did not affect copper transport and cuproptosis

**Fig. 6:**
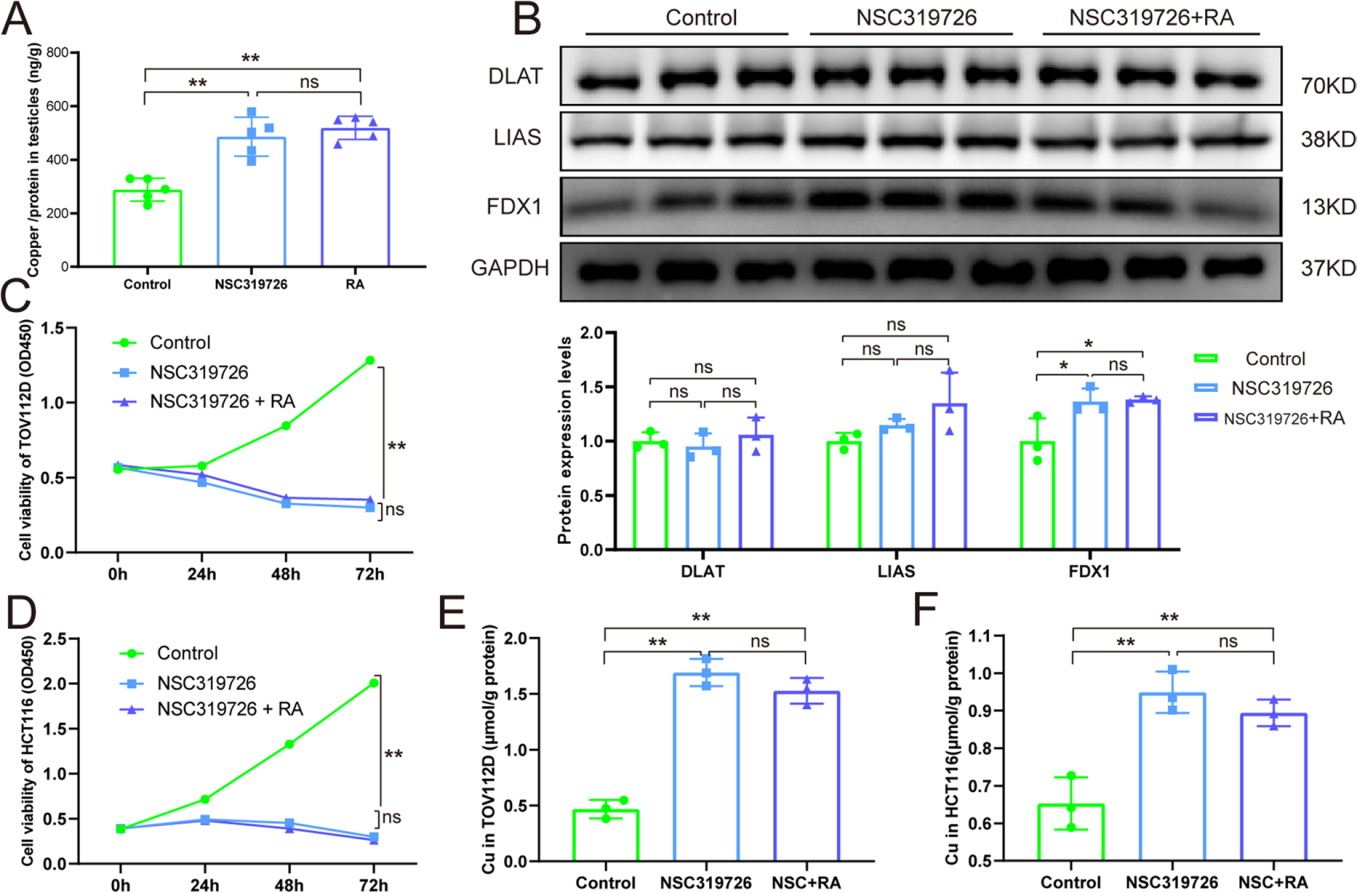
Retinoic acid did not affect copper transport and cuproptosis. (**A**) Copper levels in testes (n = 5). (**B**) Western blot analysis of DLAT, LIAS, and FDX1 in cuproptosis (n = 3). (**C**) Effects of NSC319726 and retinoic acid on TOV112D cell viability over time. (**D**) Effect of NSC319726 and retinoic acid on HCT116 cell viability over time. (**E**) Effect of NSC319726 and retinoic acid on intracellular copper ions in TOV112D cells. (**F**) Effect of NSC319726 and retinoic acid on the intracellular copper ions in HCT116 cells. *P < 0.05, **P < 0.01, and ***P < 0.001. ns, not significant

The different biological activities of NSC319726 are primarily dependent upon its ion chelating and transporting properties, which have potential cancer applications. In this study, the animal experiments showed that retinoic acid could not rescue the elevated copper ions levels in the testis (Fig. 6A) as well as the expression of crucial protein FDX1 in cuproptosis (Fig. 6B). To further determine whether retinoic acid administration could affect copper transport and cuproptosis, validation experiments were performed in cancer cells. NSC319726 reduced the cell survival of HCT116 and TOV112D cells over time, and this effect was not reversed by retinoic acid (Fig. 6C and 6D). NSC319726 increased intracellular copper ions, which were not affected by the administration of retinoic acid in HCT116 and TOV112D cells (Fig. 6E and 6F).

## Discussion

Copper ionophore NSC319726 has recently garnered significant interest in cancer treatment because of its involvement in cuproptosis. The current study first explored the potential male reproductive toxicity of NSC319726. After five weeks of NSC319726 treatment initiation, the testes and epididymis of male mice developed apparent injuries, including a decrease in testis index, sperm counts, and pregnancy rate as well as elevated sperm abnormality rate and structural disorder of testes and epididymis. Subsequent experiments indicated a decrease in total germ cells, spermatocyte, and testosterone levels and a disrupted blood-testis barrier after the NSC319726 treatment. Additionally, the concept was reinforced by an enrichment analysis of distinct metabolites and DEGs across the groups. The NSC319726 treatment downregulated CYP17A1, ZO-1, Vinculin, and β-catenin protein expression levels for the decrease in testosterone level and disrupted blood testis barrier. NSC319726 served multiple pharmacological functions in the body. The adverse impacts of NSC319726 on the male reproductive system were thoroughly investigated. However, the toxicity effects of NSC319726 on other organs require further research.

As copper ionophore NSC319726 can transport copper ions inside the cells, the NSC319726 treatment significantly increased the copper ions levels and protein level of FDX1, an essential cuproptosis-related protein, in the testis. To investigate whether the NSC319726-induced spermatogenesis dysfunction was caused by cuproptosis, a copper chelator TTM was used to treat the disorder in NSC319726-induced mice. The results indicated that TTM could partly rescue the NSC319726-induced spermatogenesis dysfunction and reduce the expression of FDX1 protein, which suggested the critical role of cuproptosis in the spermatogenesis dysfunction caused by NSC319726.

The transcriptomic data were analyzed to identify the enrichment of retinoic acid metabolism. It was found that retinoic acid levels and the expression of RDH10 protein decreased significantly in the mice testes after NSC319726 treatment. It was speculated that NSC319726 could reduce the level of RDH10, which prevented retinol from converting to retinoic acid, inhibiting the initiation of meiosis and spermatogenesis dysfunction. Moreover, retinoic acid supplementation could also improve the NSC319726-induced spermatogenesis dysfunction. Several possible explanations for the effects of NSC319726 on the expression and enzyme activity of RDH10 were proposed. NSC319726 might repress the transcription and translation of RDH10 or interact with the RDH10, thereby reducing enzyme activity. Additional research is required to investigate the fundamental molecular pathways.

Retinoic acid (RA), an active ingredient of vitamin A, plays crucial roles in numerous developmental processes in male gametes. It is synthesized from retinol in two-step oxidation by RDH10 and the aldehyde dehydrogenase 1A (ALDH1A) enzyme family (Teletin et al. 2019). The spermatogonia has three classes of cells: stem cells (self-renewing), undifferentiated spermatogonia (termed 'Aaligned,' which are part of progenitors), and differentiating spermatogonia (called 'A1', which are dedicated to spermatogenesis). Retinoic acid can induce cells to enter meiosis by transitioning Aaligned spermatogonia into A1 spermatogonia (Zhang et al. 2023). NSC319726 inhibited the expression of RDH10, preventing retinol from converting to retinoic acid and leading to the inability to initiate meiosis. The blood-testis barrier is a tight connection composed of tight junction and gap junction proteins between neighboring Sertoli cells and seminiferous epithelium cells. Retinoic acid promotes the transition of Sertoli cells to functionally differentiated cells, forming tight junctions (Nicholls et al. 2013). Retinoic acid signaling acts as an essential regulator of BTB disassembly and reassembly, and blocking the retinoic acid signaling can cause blood-testis barrier damage and steroidogenic disorder and decrease the advanced germ cells (Jauregui et al. 2018; Zhou and Wang 2022). In this study, the downregulation of the androgen synthesis protein (CYP17A1) and tight junction protein gene of BTB (ZO-1, Vinculin, and β-catenin) after the NSC319726 treatment further corroborated that NSC319726 prevented retinol from converting to retinoic acid. The NSC319726-induced retinoic acid shortage resulted in impaired spermatogenesis, reduced testosterone production, and compromised blood-testis barrier stability. NSC319726, as a retinoic acid biosynthesis inhibitor, might become a novel treatment for male contraception (Thirumalai and Amory 2021).

The different biological activities of NSC319726 are primarily dependent upon its ion chelating and transporting properties, which have potential cancer applications (Nagamori et al. 2011; Nicholls et al. 2013; Nora et al. 1977; O'Day et al. 2009; O’Brien et al. 2023; Petersen et al. 2011; Sadaka et al. 2018; Sakashita et al. 2022; Schleif et al. 2022; Seed et al. 1996). Validation experiments were performed in cells to determine whether retinoic acid administration affected copper transport and cuproptosis. The experimental results showed that the application of retinoic acid did not affect the ion chelating and transporting properties of NSC319726, nor the level of intracellular cuproptosis. Retinoic acid provided a suitable therapeutic intervention for the NSC319726-induced spermatogenesis dysfunction and did not affect its pharmacological activities.

**Fig. 7:**
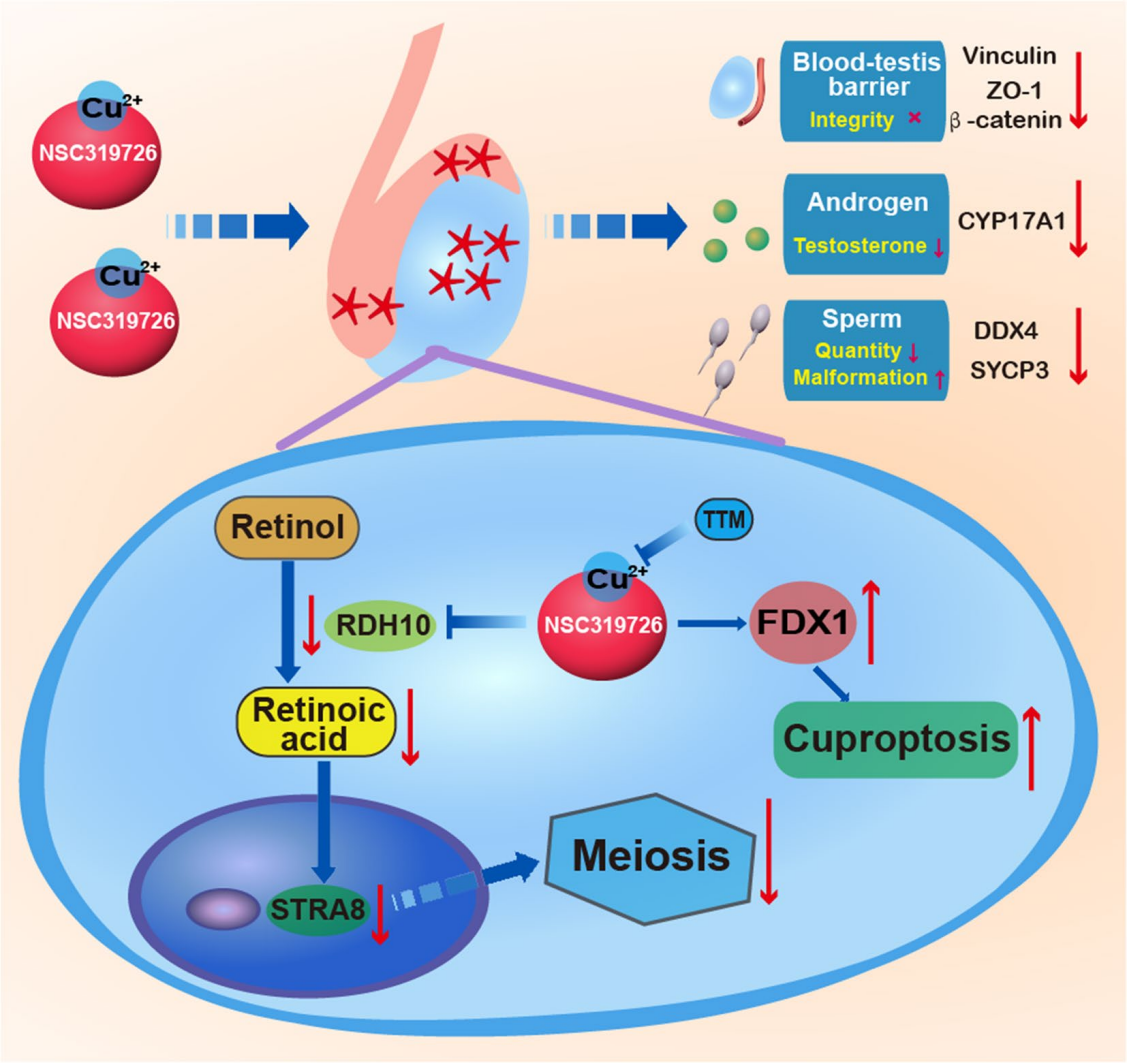
Schematic diagram of NSC319726-induced male reproductive system injury in mice. NSC319726 elevated the copper ions in the testis to induce cuproptosis and reduced the level of retinoic acid in the testis to inhibit the initiation of meiosis. NSC319726 also reduced sperm quality, decreased androgen synthesis, and disrupted the integrity of the blood–testis barrier

For the first time, the fundamental mechanisms of the toxicity of the copper ionophore NSC319726 to the male reproductive system were clarified in the current study. Results showed that NSC319726 exposure elevated copper ions in the testis to induce cuproptosis and spermatogenesis dysfunction. NSC319726 exposure also caused the downregulation of RDH10, which inhibited the conversion of retinol to retinoic acid to stimulate the initiation of meiosis. NSC319726 exposure not only harmed sperm production but also decreased germ quantities, inhibited testosterone production, and compromised the blood-testis barrier (Fig. 7). Therefore, retinoic acid can potentially be used to treat the spermatogenesis dysfunction caused by NSC319726 clinically.

## Conclusions

NSC319726 exposure can damage spermatogenesis, androgen synthesis, and the blood testis barrier, affecting the male reproductive system. Mechanistically, NSC319726 exposure decreased the protein expression of RDH10, inhibiting retinol conversion to retinoic acid. NSC319726 could also elevate the copper ions in the testis to induce cuproptosis and reduce the level of retinoic acid in the testis to inhibit the initiation of meiosis. These findings revealed the relation between retinoic acid and spermatogenesis dysfunction caused by NSC319726. This study provided a strong rationale for the clinical applications of NSC319726 in combination with retinoic acid to relieve the NSC319726 toxicity to the male reproductive system.

## Supplementary Information

Below is the link to the electronic supplementary material.

Supplementary file1 (DOCX 247 KB)

## Data Availability

All data generated or analysed during this study are included in the article. Further inquiries can be directed to the corresponding authors.
