# Supplementary material for: Retinoic acid mitigates the NSC319726-induced spermatogenesis dysfunction through cuproptosis-independent mechanisms
Source: Cell Biol Toxicol. 2024 May 1;40(1):26. doi: 10.1007/s10565-024-09857-6 (PMC11062974; doi:10.1007/s10565-024-09857-6)
Supplement: Supplementary file 1 — Supplementary file1 (DOCX 247 KB) [file 10565_2024_9857_MOESM1_ESM.docx]

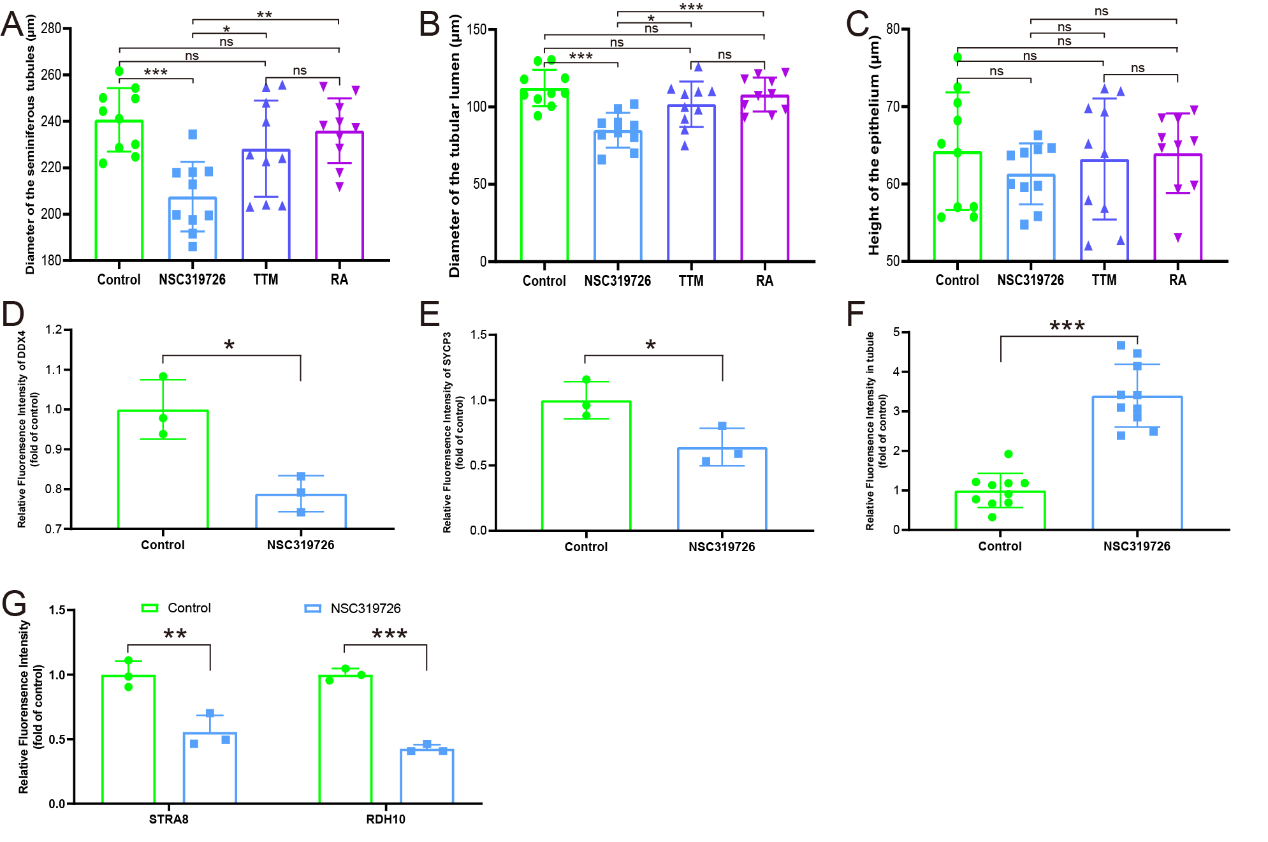


Figure S1：Quantification of HE slicing and fluorescence. (A) Diameter of the seminiferous tubules (µm). (B) Diameter of the tubular lumen (μm). (C) Height of the epithelium (μm). (D) Relative Fluorensence Intensity of DDX4 (fold of control). (E) Relative Fluorensence Intensity of SYCP3 (fold of control). (F) Relative Fluorensence Intensity in tubule (fold of control). (G) Relative Fluorensence Intensity of STRA8 and RDH10 (fold of control).
